# Factors Underlying Ebola Virus Infection Among Health Workers, Kenema, Sierra Leone, 2014–2015

**DOI:** 10.1093/cid/ciw327

**Published:** 2016-05-18

**Authors:** Mikiko Senga, Kimberly Pringle, Andrew Ramsay, David M. Brett-Major, Robert A. Fowler, Issa French, Mohamed Vandi, Josephine Sellu, Christian Pratt, Josephine Saidu, Nahoko Shindo, Daniel G. Bausch

**Affiliations:** 1 Department of Pandemic and Epidemic Diseases, World Health Organization, Geneva, Switzerland; 2 Epidemic Intelligence Service, US Centers for Disease Prevention and Control, Atlanta, Georgia; 3 Special Programme for Research and Training in Tropical Diseases, World Health Organization, Geneva, Switzerland; 4 University of St Andrews Medical School, Fife, Scotland; 5 Infectious Diseases Directorate, Naval Medical Research Center, Silver Spring, Maryland; 6 Department of Medicine and Interdepartmental Division of Critical Care Medicine, University of Toronto, Ontario, Canada; 7 Kenema Government Hospital, Kenema District, Sierra Leone

**Keywords:** Ebola, health worker, viral hemorrhagic fever, outbreak, infection prevention and control

## Abstract

***Background.*** Ebola virus disease (EVD) in health workers (HWs) has been a major challenge during the 2014–2015 outbreak. We examined factors associated with Ebola virus exposure and mortality in HWs in Kenema District, Sierra Leone.

***Methods.*** We analyzed data from the Sierra Leone National Viral Hemorrhagic Fever Database, contact tracing records, Kenema Government Hospital (KGH) staff and Ebola Treatment Unit (ETU) rosters, and burial logs.

***Results.*** From May 2014 through January 2015, 600 cases of EVD originated in Kenema District, including 92 (15%) HWs, 66 (72%) of whom worked at KGH. Among KGH medical staff and international volunteers, 18 of 62 (29%) who worked in the ETU developed EVD, compared with 48 of 83 (58%) who worked elsewhere in the hospital. Thirteen percent of HWs with EVD reported contact with EVD patients, while 27% reported contact with other infected HWs. The number of HW EVD cases at KGH declined roughly 1 month after implementation of a new triage system at KGH and the opening of a second ETU within the district. The case fatality ratio for HWs and non-HWs with EVD was 69% and 74%, respectively.

***Conclusions.*** The cluster of HW EVD cases in Kenema District is one of the largest ever reported. Most HWs with EVD had potential virus exposure both inside and outside of hospitals. Prevention measures for HWs must address a spectrum of infection risks in both formal and informal care settings as well as in the community.

## Abstract (precis)

Health workers infected with Ebola virus in Kenema, Sierra Leone, had potential virus exposure inside and outside of hospitals. Prevention measures must address a spectrum of risk factors both in formal and informal care settings as well as in the community.

The 2014–2015 Ebola virus disease (EVD) outbreak in West Africa is the most widespread in history [1]. During EVD outbreaks, health workers (HWs) are at significant risk of EVD infection because, in addition to community exposures, they carry risk of exposure during patient care. Nosocomial transmission has led to major morbidity and mortality in prior and current EVD outbreaks [2–9]. Through 1 July 2015, the World Health Organization (WHO) reported 874 cases of EVD with 509 deaths (case fatality ratio 58%) in HWs in West Africa, including 305 cases and 221 deaths in Sierra Leone [10].

The first EVD case in Sierra Leone was reported in May 2014 in Kailahun District, which shares borders with Guinea and Liberia; EVD spread to neighboring Kenema District in June 2014. Kenema Government Hospital (KGH), with support from the WHO, was the only facility in the country that provided care to EVD patients at the onset of the outbreak. KGH attended primarily to patients from Kenema District and the southern half of Kailahun District, but received cases from all other areas of the country as the outbreak progressed. A second Ebola treatment unit (ETU) managed by the nongovernmental organization Médecins Sans Frontières was established in Kailahun in July 2014 [11].

KGH is a 350-bed regional hospital covering a catchment area of approximately 670 000 people [12]. Based on an employee roster, KGH has 472 staff and volunteers. Prior to this outbreak, KGH was comprised of surgical, adult medicine, pediatric, and maternity wards, as well as human immunodeficiency virus/AIDS and tuberculosis specialty clinics. KGH has also served as the national referral center for Lassa fever, which is hyperendemic in eastern Sierra Leone [13]. A 25-bed dedicated Lassa ward divided into rooms with 2–4 beds each has been variably maintained at KGH since the 1970s, and a specialized diagnostic laboratory was established in 2004 [14]. At the onset of the EVD outbreak in Sierra Leone, the Lassa ward and laboratory were comprised of <20 people, including a doctor, nurses, laboratory technicians, and surveillance officers with extensive experience in the diagnosis and medical care of Lassa fever patients. The Lassa ward was subsequently converted into what initially was the country's only ETU. Forty-one (9%) of KGH staff worked in the ETU, augmented by 21 international workers.

As the number of EVD patients increased, KGH established 3 additional makeshift ETU wards for confirmed and suspected EVD cases, eventually totaling approximately 100 beds. Despite this, the ETU became overrun, ultimately resulting in a dangerously low HW-to-patient ratio, depletion of personal protective equipment (PPE), and inconsistent supplies of water and electricity. The situation was further exacerbated by an HW strike at KGH over delayed hazard pay starting 20 June 2014 (Figure 1), at times resulting in just a few HWs to manage up to 100 EVD patients [15]. KGH remained the largest ETU in Sierra Leone until 15 September 2014, when the International Federation of Red Cross and Red Crescent Societies (IFRC) opened a second ETU in Kenema District.

**Figure 1.**
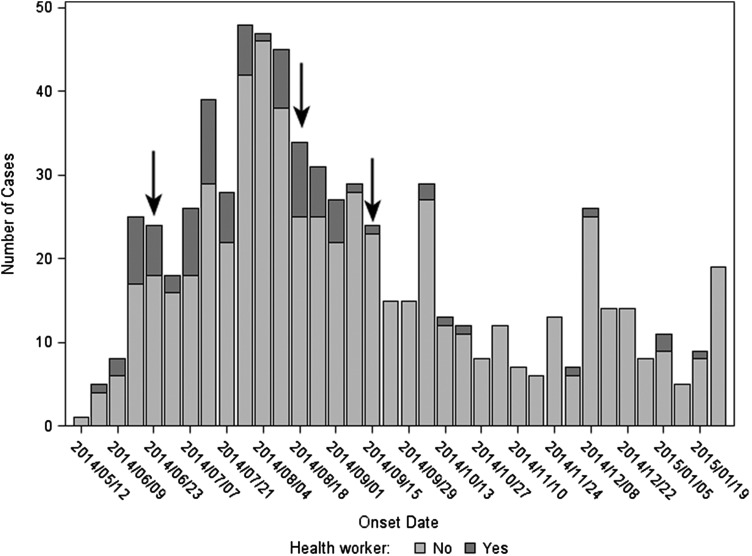
Epidemic curve for cases of Ebola virus disease, Kenema District, Sierra Leone, 1 May 2014–31 January 2015. Arrows (from left to right) indicate the beginning of the health worker strike, implementation of the new triage system at Kenema Government Hospital, and the opening of the International Federation of Red Cross Ebola Treatment Unit on the outskirts of Kenema town.

Despite the previous experience with Lassa fever patients, an unusually high number of Ebola virus infections and deaths were reported among HWs at KGH and in Kenema District [7]. To better understand how HWs became infected, as well as factors associated with infection, we explored potential sources of exposure and clinical variables of EVD in HWs in Kenema District, with a focus on KGH.

## METHODS

### Study Design

We analyzed data for suspected, probable, and confirmed EVD cases in HWs in Kenema District between 1 May 2014 and 31 January 2015 [16]. For comparison, we also included cases in non-HWs in Kenema District, as well as cases that were transferred to KGH for treatment from other districts in Sierra Leone during this period. We excluded persons <18 years of age to allow appropriate comparison between HWs and non-HWs. We also excluded cases that did not meet the WHO case definition for EVD [16]. We defined an HW as anyone who worked in a healthcare facility or engaged in healing practices (eg, traditional healers) and clinical staff as persons who have traditional patient-care roles and routinely have direct contact with patients (eg, doctors, nurses, and laboratory technicians). For non-HWs, for whom we often had incomplete data, missing dates were inferred in a similar manner as previously described [1]. For HWs, for whom more complete data were available, where dates of symptom onset or death were missing, they were imputed based on the addition or subtraction of 12 days, which was the average time from symptom onset until death in fatal cases in HWs with available dates. Laboratory confirmation of EVD was performed following established protocols [17, 18].

### Data Collection

The primary data source used was the Viral Hemorrhagic Fever database, which is the national EVD database maintained by the Sierra Leone Ministry of Health and Sanitation and consists of demographic and epidemiological as well as limited clinical data. For HWs, we supplemented this database with contact tracing records to obtain additional information about contacts with a known or suspected case, hospital staff and ETU rosters to identify and/or confirm status as an HW, and hospital records, burial logs, and public obituaries at KGH to determine outcomes. The Kenema District Health Management Team and Ebola Response Task Force approved the collection, analysis, and reporting of anonymous data as part of the outbreak response efforts.

### Statistical Analysis

We compared characteristics of HWs and non-HWs using χ^2^ tests for categorical data and *t* tests for continuous variables. We performed univariate and multivariable logistic regression models using SAS software, version 9.4 (SAS Institute, Cary, North Carolina) to estimate odds ratios (ORs) for associations between potential risk factors for EVD and death with 95% confidence intervals (CIs). Variables that were significant in univariate analysis were evaluated in multiple logistic regression models, while retaining biologically relevant variables. *P* values of <.05 were considered to indicate statistical significance.

## RESULTS

For the study period there were 706 suspected, probable, and confirmed EVD cases originating in the KGH catchment area of Kenema District and southern Kailahun or brought to KGH from other parts of the country. All 14 districts of Sierra Leone were represented. Of the 600 (85%) cases originating in Kenema District, 92 (15%) were HWs, of whom 66 (72%) worked at KGH, 17 (18%) at 8 other non-ETU health facilities in the district, and 9 (10%) unknown (Table 1). Of the 66 HWs with EVD at KGH, 58 (88%) held clinical positions, but only 18 (27%) worked in the ETU. EVD was diagnosed in 18 of 62 (29%) KGH ETU staff vs 48 (58%) of the estimated 83 clinical staff working elsewhere in the hospital.

**Table 1.** Occupations and Employment Facilities of 92 Health Workers With Ebola Virus Disease in Kenema District, Sierra Leone, 1 May 2014–31 January 2015

Characteristic	No. (%^a^)
Occupation (n = 2)	
Clinical	78 (85)
Nurse/nursing aid/state enrolled community health nurse	40 (43)
Laboratory technician	13 (14)
Maternal child health/traditional birth attendant	10 (11)
Doctor	4 (4)
Traditional healer	4 (4)
Community health officer/worker	3 (3)
Social worker	1 (1)
Vaccinator	1 (1)
Ward supervisor	1 (1)
Burial worker	1 (1)
Nonclinical	14 (15)
Administrative/supportive^b^	6 (7)
Transportation	4 (4)
Other^c^	4 (4)
Facility (n = 92)	
Kenema Government Hospital	66 (72)
Other facility in Kenema District (non-ETU)	17 (18)
Unknown	9 (10)
Worked in Kenema Government Hospital ETU (n = 66)	
Yes	18 (27)
No	48 (73)

Abbreviation: ETU, Ebola treatment unit.

^a^ Cumulative percentage does not total 100% due to rounding.

^b^ Includes cleaners, clerks, dispensers, and security.

^c^ Social worker, student/volunteer, and unknown.

HWs were similar to non-HW cases of EVD with regard to age and sex (Table 2). EVD in HWs was almost 8 times more likely to be laboratory confirmed (as opposed to probable or suspected), likely reflecting HWs' greater knowledge regarding symptoms of EVD, acknowledgment of being in a high-risk group, and ready access to laboratory testing. HWs were 2.5 times more likely to report fever than non-HWs, again probably reflecting greater self-monitoring. HWs were significantly more likely than non-HWs to identify prior contact with someone with EVD (42% vs 24%, respectively; OR, 2.9 [95% CI, 1.7–5.0]). Only 13% of the HW contacts with persons with EVD were with patients, while 27% were with other sick HWs. HWs were half as likely to report contact with sick family and relatives (43% vs 80% for HWs and non-HWs, respectively; OR, 0.2 [95% CI, .09–.5]). Although not statistically significant, HWs were half as likely to have touched a body at a funeral compared with non-HWs.

**Table 2.** Demographic Characteristics, Case Classification, Symptoms, and Type of Contact Comparison Between Health Workers and Non–Health Workers in Kenema District, Sierra Leone, 1 May 2014–31 January 2015

Characteristic	HW, No. (%)	Non-HW, No. (%)	Univariate Odds Ratio (95% CI)
Age, y, median (IQR) (excluding those <18 y old)*	39.5 (30–50)	35.0 (25–50)	…
Sex			
Female	48/92 (52)	290/614 (47)	1.2 (.8–1.9)
Male	43/92 (47)	319/614 (52)	0.8 (.5–1.3)
Unknown	1/92 (1)	5/614 (1)	1.3 (.2–11.6)
Case classification			
Confirmed	86/93 (93)	398/614 (65)	**7.8 (3.3–18.1)**
Probable	0/93 (0)	7/614 (1)	**0.9 (.8–.9)**
Suspected	6/93 (7)	209/614 (34)	**0.1 (.1–.3)**
Symptoms			
Fever	66/76 (87)	380/526 (72)	**2.5 (1.3–5.1)**
Diarrhea	29/73 (40)	206/503 (40)	1.0 (.6–1.6)
Vomiting	29/73 (40)	205/507 (60)	1.0 (.6–1.6)
Fever, diarrhea, or vomiting	73/80 (91)	432/548 (79)	**2.8 (1.3–6.2)**
Reported contact with case of Ebola virus disease	39/92 (42)	145/614 (24)	**2.9 (1.7–5.0)^a^**
Type of contact			
Family and relatives	13/30 (43)	109/137 (80)	**0.2 (.09–.5)**
Health workers	8/30 (27)	3/137 (2)	**16.2 (4.0–66.0)**
Patients	4/30 (13)	NA	…
Friends	3/30 (10)	18/137 (13)	0.9 (.3–2.6)
Other	2/30 (7)	7/137 (5)	1.3 (.3–6.7)
Funeral attendance	5/70 (7)	62/423 (15)	0.4 (.2–1.2)
Touched body	1/3 (33)	31/53 (58)	0.4 (.03–4.2)

Bold signifies statistically significant figures.

Abbreviations: CI, confidence interval; HW, health worker; IQR, interquartile range; NA, not applicable.

^a^ Missing data were excluded in the calculation of the odds ratio.

* *P* value comparing the means = .41.

There was no statistically significant difference in mean time to presentation (5.4 days for HWs vs 5.1 days for non-HWs; *P* = .51) or days from disease onset until death (11.1 days for HWs vs 8.9 days for non-HWs; *P* = .14). However, HWs spent significantly longer time admitted to the ETU before being discharged (25 days for HWs vs 16 days for non-HWs; *P* = .02).

Cases of EVD in HWs were identified throughout the study period. HWs represented a larger proportion of all EVD cases, sometimes up to 25%, prior to mid-September 2014, after which only sporadic cases of EVD in HWs were seen (Figure 1). This change corresponded to the implementation of a revamped triage system for patients suspected of having EVD at KGH on 19 August 2014, as well as the opening of the IFRC ETU in September 2014. Taking these 2 events together, and taking into account the maximum 21-day incubation period for EVD, cases were more likely to be HWs at KGH than non-HWs prior to implementation of the triage system and opening of the new ETU (89% vs 54%, respectively; OR, 7.1 [95% CI, 3.6–13.9]), whereas this relationship was essentially inverted after these events (11% for HWs vs 46% for non-HWs; OR, 0.1 [95% CI, .01–.3]).

Case fatality was 69% for HWs and 74% for non-HWs (*P* = .30). In the univariate analysis combining both HWs and non-HWs, factors associated with fatal EVD were age >45 years (OR, 3.4 [95% CI, 2.1–5.5]), presentation >7 days after symptom onset (OR, 0.3 [95% CI, .2–.6]), and presence of fever (OR, 2.4 [95% CI, 1.1–5.4]) (Table 3). There was no association between mortality and sex, designation as clinical or nonclinical staff, facility where worked, or working/not working in an ETU.

**Table 3.** Associations Between Demographic and Clinical Variables and Ebola Virus Disease Mortality, Kenema District, Sierra Leone, 1 May 2014–31 January 2015

Factor	No.	Univariate		Multivariable	
		OR (95% CI)	*P* Value	OR (95% CI)	*P* Value
Age					
<45	257/385	1.0	…	1.0	…
≥45	155/178	3.4 (2.1–5.5)	<.0001	2.1 (.9–5.0)	.081
Sex					
Female	196/277	1.0	…	1.0	…
Male	213/282	1.3 (.9–1.9)	.203	1.5 (.8–2.6)	.209
Time to presentation					
≤7 d	387/514	1.0	…	1.0	…
>7 d	25/49	0.3 (.2–.6)	.0004	.3 (.1–.8)	.013
Symptoms					
Diarrhea	117/176	1.1 (.7–1.9)	.681	1.3 (.7–2.6)	.415
Fever	248/353	2.4 (1.1–5.4)	.030	2.1 (.9–5.1)	.087
Vomiting	119/183	0.9 (.5–1.5)	.723	1.1 (.5–2.1)	.818

Hosmer-Lemeshow test of goodness-of-fit was performed (*P* = .916).

Abbreviations: CI, confidence interval; OR, odds ratio.

The final multivariable model included age, sex, symptoms, and time from symptom onset to ETU presentation. In this model, only presentation >7 days after symptom onset was associated with a significantly decreased risk of death (OR, 0.3 [95% CI, .1–.8]), probably because most of these patients had already passed the mean time to death for EVD, which is usually around 8–10 days [19, 20]. The odds of death in persons ≥45 years old was >2 times that of younger people, although this result was no longer statistically significant (OR, 2.2 [95% CI, .9–5.0]).

## DISCUSSION

We describe one of the largest clusters of EVD among HWs ever reported. Eighty EVD cases in HWs were noted in the 1995 outbreak in Kikwit, Democratic Republic of the Congo, from various health centers [2]. Most HWs with EVD in Kenema had numerous risk factors for virus exposure in ETUs, other areas of the hospital, and in the community, making it difficult to ascertain where Ebola infection occurred. Furthermore, informal discussions with many of the KGH HWs with EVD revealed no discrete infecting events, such as needle-sticks or fluid splashes to mucous membranes, suggesting that such events were not central to the high attack rates in this group.

In contrast to the Ebola virus outbreak in Kikwit, HW infections continued to occur in Kenema even after the creation of the ETU and enhanced infection prevention and control (IPC) measures, including provision of PPE. The large number of Ebola virus infections in HWs at KGH seems all the more surprising because, prior to the outbreak, KGH might have reasonably been considered to be one of the best-prepared hospitals in West Africa to care for EVD patients, considering the long experience with Lassa fever [14]. Previous training and experience caring for Lassa fever may have indeed helped protect HWs who worked in the ETU, perhaps explaining the lower EVD incidence in this group relative to those who worked outside the ETU.

With regard to possible exposures in the Lassa ward-turned-ETU, we speculate that various underlying factors with antecedents long before the beginning of the EVD outbreak may have resulted in a high-risk environment for HWs. In recent decades, the KGH Lassa fever program has been primarily laboratory focused [14, 21]. Patient care aspects have been consistently underresourced, with the Lassa ward itself in need of significant renovation and the reinforcement of appropriate IPC practice, and supply of PPE inconsistent and piecemeal [21]. Somewhat indicative of these suboptimal conditions is the observation that Lassa virus infection frequently occurred in Lassa ward staff, indicative of less-than-ideal IPC practices [14, 21, 22]. A considerable number of Lassa ward staff may have been immune to Lassa fever from previous exposure either in the clinical setting or in the community, giving a false sense of security with regard to the efficacy of current IPC practices [21]. It should also be noted that the infectious dose of Ebola virus is considered to be lower than that of Lassa virus [23, 24].

As the peak of the EVD outbreak hit eastern Sierra Leone, KGH ETU became the major referral center for Kenema District, southern Kailahun district, and all other areas of Sierra Leone. ETU staff were overburdened, caring for a number of patients well beyond their capacity, without sufficient staff to always work in pairs, as is recommended to ensure IPC practices, including safe donning, doffing, and decontamination. IPC practices often quickly deteriorate under such circumstances, somewhat independent of staff experience, leading to an increase in infection risk [25]. This dangerous situation was further exacerbated by the KGH HW strike [15] and the loss of HWs to EVD, including the doctor and nurse in charge of the KGH ETU, which further decreased numbers and morale [26].

As often noted in hemorrhagic fever outbreaks, HWs were among the first documented cases of EVD in Kenema (Figure 1) [2, 4, 27]. Monitoring of HWs as a sentinel group may be a logical strategy for early detection in nonaffected regions. A sharp decline in the number of EVD in HWs was noted after the implementation of a revamped triage system at KGH, a finding noted with the implementation of similar systems in previous Ebola outbreaks [28–30] and indicative of the nonspecific clinical presentation of EVD that makes distinction from many other common febrile diseases difficult [31]. The new triage system, designed and built in collaboration with the IFRC, served as the single point of entry to KGH for all patients and consisted of a unidirectional, 2-stage process to more thoroughly evaluate patients, improve patient flow, and minimize cross-contamination resulting from overcrowding. Whereas the previous triage system was managed by laboratory technicians in a small tent, the new system consisted of nurses and cleaners trained and supervised by the IFRC. After the new KGH triage was established, 700 persons were screened and 69 patients were admitted to the ETU during the first week alone (S. Boye, personal communication, 2014). The opening of the IFRC ETU on the outskirts of Kenema town several weeks later further reduced the burden of cases at the KGH ETU although, as only a few patients were admitted to the IFRC facility per week for the first few weeks of operation, the impact was probably modest. It should also be noted that the HW strike that began in June 2014 and persisted on and off for months resulted in diminished numbers of HWs entering the ETU, and thus diminished risk from that source.

Although there were numerous vulnerabilities in the patient care setting at KGH, both inside and outside the ETU, it would be a mistake to focus exclusively on the hospital. Clearly, many cases of Ebola infection in HWs were acquired elsewhere, as evidenced by the fact that almost three-quarters of the cases occurred in personnel who did not work in the ETU, including in nonclinical staff. These findings are consistent with studies in Liberia, Sierra Leone, and Uganda where HWs in non-ETU settings and those without traditional patient care roles frequently became infected [6, 8, 9, 32]. HWs are, after all, members of the community, sharing many of the same transmission risks from contact with family, relatives, and friends (Table 2). In addition, it was widely suspected and anecdotally reported that many HWs cared for patients outside of normal working hours and settings, often without full PPE. The desire of HWs to care for sick colleagues may pose a particular risk, one for which compassion may at times supersede safety. In one instance, an HW, who later died of EVD, was found in the ETU attempting to start an intravenous line on an admitted colleague with EVD, gloves being the only PPE worn. When noticed and questioned the only response was “I am trying to help my colleague!” Another HW at KGH was exposed and infected while taking care of an orphan outside of an ETU when the child became symptomatic [15].

Our study is subject to several limitations: (1) Although not part of the study, we spoke with HWs to ascertain their exposures whenever possible. However, because of its retrospective nature, we were unable to interview all HW cases. (2) We could not determine from the available data whether HW's contact with persons with EVD was protected or unprotected, or whether breaches in protocol may have occurred, and thus cannot make conclusions regarding the efficacy or inefficacy of any given PPE or IPC measure. (3) We likely underestimated the number of HWs in the community in Kenema (ie, those who did not work at KGH). In addition, we used a broad definition of HW, which included nontraditional patient care roles, and some persons who fit this definition may not have self-identified as HWs when interviewed. (4) The only clinical data available to us were the presence of fever, diarrhea, and vomiting, hindering an in-depth analysis of the relationship between clinical presentation and disease evolution. (5) Data on HWs, especially those who developed EVD, may have been more thoroughly recorded than data on non-HWs, potentially confounding analyses of potential exposures and disease outcomes between the 2 groups.

The large cluster of EVD cases among HWs in Kenema District emphasizes the high burden of disease in HWs in the Ebola outbreak in West Africa, with important implications for prevention. While much attention is rightly paid to IPC measures specific to the ETU, HWs may incur considerable risk in other areas of the hospital, especially given the difficulty of making a clinical diagnosis of EVD, as well as in their communities, where they may continue to formally or informally practice their profession. IPC measures must therefore address a spectrum of risk factors related to formal care settings in the ETU, as well as hospital-wide, and to informal patient care and social settings in the community.
